# Exercise Trajectories of Women from Entry to a 6-Month Cardiac Rehabilitation Program to One Year after Discharge

**DOI:** 10.1155/2013/121030

**Published:** 2013-09-12

**Authors:** Heather M. Arthur, Chris Blanchard, Elizabeth Gunn, Jennifer Kodis, Steven Walker, Brenda Toner

**Affiliations:** ^1^Faculty of Health Sciences, McMaster University, 1280 Main Street West, Hamilton, ON, Canada L8N 3Z5; ^2^Hamilton Health Sciences, 237 Barton Street East, Hamilton, ON, Canada L8L 2X2; ^3^Dalhousie University, 5790 University Avenue, Halifax, NS, Canada B3H 1V7; ^4^Niagara Health System, 155 Ontario Street, 2nd Floor, St. Catherines, ON, Canada L2R 5K3; ^5^University of Toronto, 1 King's College Circle, Room 2370,Toronto, ON, Canada M5S 1A8

## Abstract

*Background*. Physical activity is associated with reduced mortality and morbidity. Cardiac rehabilitation (CR) is an effective intervention for patients with cardiovascular disease (CVD). Unfortunately, women are less likely to engage in, or sustain, regular physical activity. Objectives were to (1) describe women's guidelines-based levels of physical activity during and after CR and (2) determine the physical activity trajectories of women from entry to CR to one year after CR. *Methods and Results*. A prospective, longitudinal study of 203 women with CVD enrolled in a 6-month CR program. Physical activity was measured using the Godin Leisure Time Exercise Questionnaire (LSI), focusing on moderate-strenuous activity. Data were analyzed using latent class growth analysis (LCGA) and logistic regression. Mean scores on the LSI showed women to be “active” at all follow-up points. LCGA revealed a two-class model, respectively, called “*inactive relapsers*” and “*moderately active relapsers*.” Predictors of the “*moderately active relapsers*” class were employment status and diagnosis of myocardial infarction. *Conclusions*. Women achieved the recommended physical activity levels by the end of CR and sustained them until one year after CR. LCGA allowed us to determine the class trajectories associated with moderate-strenuous activity and, from these, to identify implications for targeted intervention.

## 1. Introduction

Cardiovascular disease (CVD) is a leading cause of death among North American women and is recognized as a major women's health issue [1, 2]. Although mortality rates have declined, the actual number of deaths due to CVD has increased. This has been attributed largely to an aging population, and, as women tend to live longer than men, it is expected that the number of deaths from CVD may one day be higher in women than in men [3]. Mortality data figure prominently in the scientific literature; however, the morbidity of CVD is also of great importance. In Canada, 25% of adults over 70 years of age report having heart problems which affect or impair their lives by causing them to feel less healthy, require assistance with normal activities of daily living, and restrict activity [1].

Physical activity has been strongly associated with health and health related quality of life (HRQL) [4]. In the recently published Global Burden of Disease Study 2010 [5], physical inactivity and low physical activity accounted for 3.2 million (2.7 million to 3.7 million) deaths and 2.8% (2.4 to 3.2) of disability-adjusted life years in 2010. Furthermore, cardiac rehabilitation (CR) has been shown to be an effective intervention and is considered as an essential element in the continuum of care for patients with CVD [6–10]. A recent Cochrane review confirmed a 26% (RR = 0.74 (95% CI 0.63, 0.87%)) reduction in cardiovascular mortality with exercise-based CR [11]. More recently, Martin et al. [12] reported that the completion of a CR program was associated with a lower risk of death, a lower risk of all-cause hospitalization, and a lower risk of cardiac hospitalization. Published outcomes of participation in CR include improvements in physical functioning and risk factor profile as well as decreased activity-related symptoms and disability. 

Structured exercise is the cornerstone of CR programs, where exercise prescriptions are based on the joint scientific statement from the American Heart Association (AHA) and the American Association of Cardiovascular and Pulmonary Rehabilitation (AACVPR) and include recommendations for daily exercise [13]. A structured physical activity program is designed to enhance exercise capacity which, as mentioned, has consistently been shown to be an independent predictor of survival, particularly among men. Despite the benefits associated with participating in CR [10], less than 55% of patients meet the moderate-strenuous physical activity recommendations during CR [14, 15] and less than 50% meet these recommendations three months after completing CR [16, 17]. Many patients may not be engaging in sufficient physical activity to garner the benefits, and unfortunately, women, older adults, and those who are socially disadvantaged have been described as the least likely to engage in regular physical activity [18, 19]. Thus, we must meet the challenge to engage and sustain women's involvement in exercise and regular physical activity. 

The long-term success of CR rests in part on the patient's ability to maintain health related behaviours, including regular exercise, following the end of formal treatment. Very little data exist related to women's ability to achieve moderate-strenuous activity levels during CR and sustain them beyond discharge from formal programs. In order to meet the increased demand from both patients and their primary care providers for effective services and to preserve documented short-term benefit from participation in secondary prevention programs, data on the outcomes in women, such as longer term adherence with physical activity recommendations, are required. 

## 2. Purpose 

There were two study objectives: (1) to describe women's guidelines-based levels of physical activity at the conclusion of a formal CR program and (2) to determine the physical activity trajectories of women from entry to CR to one year after CR. Physical activity was measured by the Godin Leisure Time Exercise Questionnaire (LSI) [20]. Measurements were taken at 4 time points: (a) entry to CR (baseline), (b) completion of a 6-month CR program, (c) 6 months after CR discharge, and (d) one year after discharge.

## 3. Methods

Women were recruited from two CR sites: the Cardiac Health and Rehabilitation Centre (CHRC) of Hamilton Health Sciences, Hamilton, Ontario, Canada, and the Cardiac Health and Rehabilitation Program (CHRP) located in the Niagara region. The CHRC and CHRP are identical; both offer a multidisciplinary, outpatient CR program, with supervised exercise classes, dietary and psychological counselling, and nursing education and support. Ninety-minute supervised exercise classes were held twice weekly over a 6-month period for a total of 48 exercise sessions. The CHRC has established itself as a viable research site with numerous clinical CR studies having been successfully conducted in this setting [21–25]; the CHRP was later created as a satellite site of the CHRC with the same program model and goal structure.

## 4. Inclusion/Exclusion Criteria

Patients were eligible to participate in the study if they (1) were females; (2) had documented heart disease; (3) could ambulate independently; (4) were over the age of 18; (5) provided informed consent; and (6) were able to read and write English. Patients were excluded if they (1) had a positive graded exercise test (defined by a drop in the systolic blood pressure greater than or equal to 20 mm Hg, symptoms of angina, or uncontrolled atrial or ventricular dysrhythmias or horizontal or downsloping ST segment displacement of 0.1 mV or more at 80 s after the J point); (2) were unable to attend rehabilitation twice a week; (3) were unable to participate due to physical limitations; and (4) had previously participated in an outpatient CR program. 

## 5. Procedures 

The study was approved by the joint Research Ethics Board of the McMaster University and Hamilton Health Sciences and the Research Ethics Board of the Niagara Health System. At the CHRC a nurse clinician contacted each patient on the referral list to review cardiovascular risks, cardiac history, and general medical history and to discuss their first visit to the center. This person asked the patient if she was willing to be contacted about a study related to women with heart disease. For those willing to be contacted, the research coordinator identified potentially eligible participants from the notes provided by the nurse. The coordinators then telephoned eligible participants to recruit them into the study. At the CHRP women were approached for possible participation in the study during their intake appointment. 

Baseline assessments included the collection of the demographic, clinical, and physical activity measures analysed in the current paper including a complete medical history, a full physical examination, and either a symptom-limited graded upright cycle ergometry exercise test with direct measurement of oxygen uptake (CHRC) or a treadmill test using the Bruce protocol (CHRP) (GXT). Exercise tests were used to develop the patient's individually tailored exercise prescriptions. Women who agreed to participate in the current study met with the research coordinator after their intake appointment at the CHRC or the CHRP to complete informed consent and the baseline measures. 

The study was initiated at the CHRC. Subsequently, funding for the research was extended by the granting agency in order to add the second site (CHRP), which had recently opened. The addition of a second site was desirable in order to increase the sample size and ultimately provide one of the largest women-only samples in CR research. Since the CHRP was added as a study site after the study had started, the length of followup at that site was reduced. Therefore, repeated assessments were conducted at the following time points: (a) baseline, conclusion of the formal 6-month CR program, and at 6 and 12 months after CR completion at the CHRC and (b) baseline, conclusion of the 6-month CR program, and 6 months after completion at the CHRP site. 

## 6. Measures

1. Demographic Characteristics and Medical Assessment.
  - The CHRC and CHRP have standard intake and posttreatment assessment procedures. Demographic assessment includes age, marital status, living arrangements, highest level of education completed and socioeconomic and employment status. A complete medical history is taken, and a risk factor profile is conducted (including blood pressure, triglycerides, total cholesterol, LDL-C and HDL-C, serum hemoglobin A1c, and smoking status). All patients undergo a GXT (as described above) under the supervision of a cardiologist for the purpose of establishing a CR exercise prescription.
2. Exercise Behaviour.
  - The criterion dependent variable in the current study was the performance and maintenance of the moderate-strenuous physical activity. Participants were assessed for their ability to achieve moderate-strenuous activity during CR and in the postrehabilitation environment. The physical activity was measured by the LSI. The LSI contains 3 questions that assess the frequency of the mild, moderate, and strenuous exercise performed for at least 15 minutes in duration during free time in a typical week. The LSI is scored as follows: the self-reported weekly frequencies for strenuous, moderate, and mild intensity levels are multiplied by 9, 5, and 3 METs (metabolic equivalents), respectively. These scores are summed, and total scores of ≥24 units are defined as “active,” whereas 23 units or less are described as “inactive” and of no health benefit [20]. An independent evaluation of this measure found its reliability and validity to compare favourably to 9 other self-report measures of exercise based on various criteria including test-retest scores, objective activity monitors, and fitness indices [26]. Additionally, the LSI has been used frequently in CR [27, 28]. According to Godin (2011) [29], the examination of physical activity from a health benefit perspective should focus only on the moderate and strenuous categories of the scale.

## 7. Sample Size and Data Analysis

We used Green's formula [30] for sample size calculation where *n* ≥ *L*/*f* ^2^. 

To calculate “*L*”, the equation *L* = 6.4 + 1.65*m − 0.05*m^2^ is applied, when the number of variables (*m*) ≤ 10. According to Green's formula, *f* ^2^ is associated with effect size, whereby a value of *f* ^2^ = 0.15 represents a medium effect size. For every variable > 10, *L* is increased by 0.6. For power estimation with multiple regression, an *α* = .05, power = .80, ES (*f* ^2^ = .15), and 12 predictor variables, we required a sample size of 135 women. Taking into account a possible attrition rate of 20%, the target sample size was 162 female CR participants. 

 The analysis plan included the following: descriptive analysis, latent class growth analysis, and logistic regression analysis. A common approach to examining exercise over time is the use of a repeated measures analysis of variance. This approach assumes that patients come from a single population and that a single growth trajectory can adequately approximate an entire population (i.e., that the growth trajectories are homogeneous). However, existing studies [31–33] have categorized patients into distinct subpopulations (e.g., via gender, age groups). These subpopulations may have their own growth trajectories that are potentially different from the overall growth trajectory (i.e., the overall growth trajectory may be heterogeneous). Latent class growth analysis relaxes the growth heterogeneity assumption and allows for differences in growth trajectories across subpopulations via latent trajectory classes [34, 35]. In the context of the current study, for a given exercise intensity, latent class growth analysis can be used to simultaneously estimate the number and size of the potential latent classes and determine which demographic/clinical covariates might predict class membership. Latent class growth analysis provides significantly more information than traditional analytical approaches in terms of identifying key target groups for physical activity intervention. 

A series of latent class growth analyses [36] were conducted in MPLUS 6.1 using maximum likelihood estimation. This approach is particularly useful for unbalanced designs because it enables all patients to be analyzed if they provide at least 1 data point in the trajectory. In terms of the analyses, a latent intercept growth factor (i.e., baseline physical activity) and a latent slope growth factor (i.e., change in physical activity over time) were created. A quadratic term was also tested for its necessity in the models. To identify the number of classes, the Bayesian Information Criterion (BIC), the Bootstrapped likelihood ratio (BLRT), and entropy indices were used. When comparing a 2-class versus single class model, for example, a change in the BIC > 10, higher entropy value (near 1.0), and a BLRT *P* value < .05 would be considered as an evidence favoring the 2-class over the single class model [37]. Once the final number of classes was generated, a series of *χ* ^2^ analyses were conducted in SPSS 20.0 to determine potential demographic and clinical differences across the classes. All significant variables were then entered into a logistic regression to delineate the most important class membership predictors.

## 8. Results

### 8.1. Patient Population

**Figure 1:**
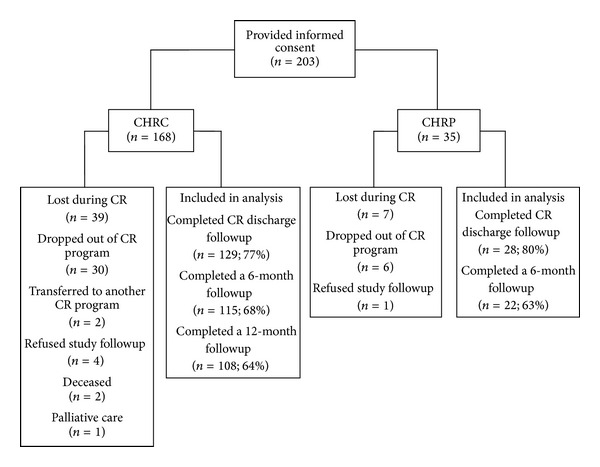
Flow of program and study participants.

A total of 203 women were recruited to the study and completed all baseline measures. As expected, there was attrition over the course of the 1.5 years following enrollment (Figure 1). At the end of CR, 157 women (77.3%) completed the LSI; at a 6-month followup 137 (67.5%) women completed the LSI. Since a one-year followup was restricted to the main study site due to inclusion of the second site at a later date, the one-year followup completion rate was 64% of the eligible sample. There were no differences in patients' characteristics between sites (Table 1). Dropout was shown to be significantly related to having an increased number of comorbidities at (a) completion of CR (*P* = .00), (b) 6 months following CR (*P* = .00), and (c) one year following CR (*P* = .00). The mean number of comorbidities for women who stayed in the study versus those who dropped out was as follows: 2.03 versus 2.67 (at completion of CR), 1.98 versus 2.57 (6 months following CR), and 1.97 versus 2.60 (one year following CR). At the 6-month followup assessment only, marital status was significantly related to dropout (*P* = .004); married women were less likely to drop out than nonmarried/nonpartnered women. 

 The majority of participants had had a percutaneous coronary intervention (PCI) (42.9%), followed by coronary artery by-pass graft surgery (CABG) (33%) and myocardial infarction (MI) (16.3%). The mean age of the participants was 63 ± 10.9 years and the majority of women had hypertension (70%) and dyslipidemia (76.8%). Most women were either retired or not working (64%) (Table 1). 

### 8.2. Physical Activity during CR

Based on the LSI, women exercised a mean number of 27 units per week by the end of CR, compared to a mean of approximately 18 units per week at entry. This difference was statistically significant (*P* < 0.01). Therefore, over the course of the CR program, women increased their physical activity from a mean score that was “inactive” to one that was “active” (≥24 units of exercise per week) and likely to be of health benefit. 

The mean number of units exercised per week at the 6-month followup was maintained at approximately 27 (*n* = 137), which is considered “active.” Since activity levels were maintained during the 6 months after discharge from CR, this finding represents a significant improvement in scores between the baseline and the 6-month followup (*P* < 0.01). 

The mean number of units exercised per week at a one-year followup was approximately 24 (*n* = 108). At a one-year followup (18 months from baseline), women were still “active.” The drop from 27 to 24 units between 6 months after discharge from CR and one year after discharge was not statistically significant.

### 8.3. Latent Class Growth Analysis

**Figure 2:**
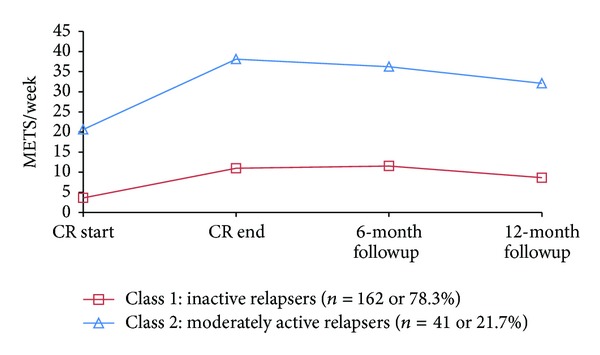
Physical activity patterns for moderate to strenuous intensity from baseline to 18 months.

As can be seen from Table 2, results indicated that the 3-class model showed the best model fit. However, within the 3 class model, the 3rd class only had 18 participants making subsequent analytical comparisons difficult. Therefore, we proceeded with a 2-class model. As shown in Figure 2, the first class (*Inactive Relapsers*) started the CR doing very little moderate-strenuous activity. While this level of activity increased during a CR, it began to decline after the 6-month followup measurement. The second class (*moderately active relapsers*) began the CR relatively active and increased their moderate-strenuous activity during the CR. After discharge from the CR the women in this class showed a gradually decreasing trajectory of moderate-strenuous physical activity by 12 months after CR, though they continued to maintain the activity levels that were between 30 and 35 METs per week. 

### 8.4. Logistic Regression Analyses

Table 3 shows that the distributions for employment status and diagnosis varied by class; therefore, they were entered into the final logistic regression. Results showed that patients were significantly more likely to be *moderately active relapsers* if they were employed versus not employed (odds ratio = 2.93, *P* < .01) and had an MI versus other diagnoses (odds ratio = 3.25, *P* < .01). 

## 9. Discussion

In one of the largest, female-only CR samples in the published literature we examined women's ability to achieve guidelines-based levels of physical activity (moderate-strenuous) during a 6-month CR program and up to one year following discharge from the program. Further, we used a latent class growth analysis to identify distinct classes of the long-term physical activity trajectories in women. We are not aware of other studies, specifically in women, where the authors have identified either the latent classes within women's exercise patterns or the trajectories that may be uncovered within them. Physical activity is not static; it is an ever changing process that is responsive to multiple factors such as motivation, intention, context, and daily circumstances, to name only a few. Most authors have tended to analyze exercise behaviour over time by averaging repeated assessments of activity. This approach does not capture fully the interplay of time and measurement points when longitudinal data are available [38]. It is important to remember that latent class growth analysis is a person-centered analysis, not the standard aggregate level type of analysis (e.g., ANOVA, linear regression). The sustained moderate-strenuous physical activity 6 months following discharge from formal CR was assessed by the LSI. At this time point approximately 68% of the original sample was retained and the data showed women to be “active”; this was a statistically significant improvement over baseline measurement of physical activity and represented no decline from the time of discharge from CR. Among those who were retained one year after the discharge from CR (approximately 64% of the CHRC group; those who were followed for one year), women were still physically “active.” It has long been known that relapse rates are high following participation in the CR programs. Typically, only 30–60% of patients who complete a CR program are still exercising 6 months later [39]. In their study that focused on women, Moore et al. [17] reported that only 28% were exercising 3 times a week, three months after CR. It is unclear whether those who were continuing to exercise in the previously mentioned studies were doing so at a level that would contribute to long-term health benefits. However, from the current study, we note that the majority of women, on average, were still exercising at the level required to achieve health benefits one year after discharge from the CR. 

The interpretation of these physical activity rates is a key reason why latent class growth analysis was chosen; uncovering classes of women whose exercise behaviour followed a different trajectory than others is more informative than simply examining average group scores. 

 Different trajectories were found regarding the moderate-strenuous physical activity classes. Two classes emerged: the *inactive relapsers* (approximately 78% of sample) and the *moderately active relapsers *(approximately 22% of sample). The down sloping trajectories of these two classes are consistent with the published literature about physical activity continuation after the CR [31]. However, despite increases in physical activity during the CR, the *inactive relapsers* were not sufficiently active throughout the entire study to achieve a health benefit. The *moderately active relapsers* improved their level of physical activity during the CR by approximately 80% from baseline and were achieving ≥24 units on the LSI (and 30–35 METs per week) both during the CR and throughout followup. 

 The *moderately active relapsers* may be an important target group for interventions aimed at sustaining physical activity levels that were clearly achievable, whereas the *inactive relapser* class requires a different intervention altogether: one that focuses on preventing a decline in activity that begins immediately following conclusion of the formal CR program. 

*Moderately active relapsers* were more likely to be employed and to have had an MI versus other cardiac diagnoses. Moore et al. [17] found comorbidity to be the only significant predictor of exercise frequency and exercise intensity in their sample of 60 women who were recruited at the completion of a formal CR program. There was no significant difference in the number of comorbidities between the two latent classes found in this study. It is possible that being employed was a contributor to the observed mild activity decline, which began 6 months after discharge from CR. For employed women who return to work, strategies to help them sustain their health-promoting level of physical activity will be paramount. 

## 10. Limitations

The findings from this study warrant replication due to some limitations. First, the prospective, observational design meant that the participants were self-selected. A randomized controlled trial is not the appropriate design to examine questions related to predictors and correlates of physical activity; no intervention is being tested. It is possible that bias was operational if the participants were particularly interested in a study of women only and were keen to help address issues of relevance to women; however, our data show that the characteristics of this sample were comparable to those in other studies of women in the CR. Second, the use of self-reported physical activity may have led to an over- or underestimation of the actual behaviour. We have previously used the gold standard exercise stress test in most of our CR research but have found that in the studies of a long-term followup, participants are less and less likely to return for repeat stress testing [40]. Thus, the advantage of using a harder measure of physical activity may be outweighed by a loss of data over time. The study included two CR sites. The delivery of CR services is generally not tightly standardized across centers; therefore a strength of this study is the fact that the service model was virtually identical at both sites, since one was opened as a satellite of the other. Furthermore, site was not found to be a predictor of physical activity trajectories, suggesting that using two sites did not bias the results in any systematic fashion. Dropout rates were lower in this study than in other comparable studies reported in the literature. Nevertheless, drop was related to being unmarried/unpartnered and to a greater number of comorbidities. This finding is very helpful for investigators doing future research with female cardiac patients as they may be able to mitigate dropout by the awareness of these factors. The findings may be generalizable to CR centers that use a similar, comprehensive model of care, but not to women with CVD who have not been referred to CR. 

## 11. Conclusion

In summary, we found that there may be two physical activity trajectories of female patients with heart disease during and after a formal CR program. Overall, the average level of physical activity at the conclusion of CR and after was high enough to achieve health benefits up to one year after discharge from CR. However, latent class growth analysis allowed us to examine which class (*moderately active relapsers*) may have explained the pooled finding in regard to physical activity. Women remain an important target for physical activity promotion since, for many, their trajectories may be unfavourable over the long term after discharge from CR. Our findings provide important direction for where and when to intervene to prevent physical activity decline in women following a cardiac event. 

## Figures and Tables

**Table 1:** Characteristics of study participants.

Variable	*N* = 203	CHRC (*n* = 168)	CHRP (*n* = 35)
Age			
Mean (SD)	63.09 (10.96)	63.00 (11.13)	63.54 (10.23)
Range	32–84	32–84	44–81
Marital status, *N* (%)			
Married	118 (58.1)	99 (58.9)	19 (54.3)
Nonmarried, living with partner	12 (5.9)	8 (4.76)	3 (8.57)
Partner, not cohabiting	1	0 (0)	1 (2.9)
Divorced/Separated	30 (14.8)	4 (2.4)	2 (5.7)
Widowed	37 (18.2)	27 (16.1)	3 (8.6)
Single	6 (3.0)	30 (17.9)	7 (20.0)
Living arrangements, *N* (%)			
With others	155 (76.4)	128 (76.2)	27 (77.1)
Alone	48 (23.6)	40 (23.8)	8 (22.9)
Education level, *N* (%)			
Did not complete high school	43 (21.2)	34 (20.2)	9 (25.7)
Completed high school	59 (29.1)	49 (29.2)	10 (28.6)
Some college or university	29 (14.3)	25 (14.9)	4 (11.4)
Completed college or university	33 (16.3)	28 (16.7)	5 (14.3)
Complete some or all graduate/professional school	39 (19.2)	32 (19.0)	7 (20.0)
Employment status, *N *(%)			
Full-time	27 (13.3)	22 (13.1)	5 (14.3)
Part-time	25 (12.3)	21 (12.5)	4 (11.4)
Unemployed	14 (6.9)	8 (4.8)	6 (17.1)
Retired	112 (55.2)	96 (57.1)	16 (45.7)
Leave of absence	5 (2.5)	4 (2.4)	1 (2.9)
Other	20 (9.9)	17 (10.1)	3 (8.6)
Referral event, *N *(%)			
Myocardial infarction	24 (11.8)	18 (10.7)	6 (17.1)
CABG	69 (34.0)	62 (36.9)	7 (20.0)
Valve replacement	4 (2.0)	2 (1.2)	2 (5.7)
PCI	97 (47.8)	80 (47.6)	17 (48.6)
CHF	1 (0.5)	1 (0.6)	0 (0)
Risk management	8 (3.9)	5 (3.0)	3 (8.6)
Hypertension, *N* (%)			
Yes	142 (70.0)	122 (72.6)	20 (57.1)
No	58 (28.6)	43 (25.6)	15 (42.9)
Unknown	3 (1.5)	2 (1.2)	0 (0)
Dyslipidemia, *N* (%)			
Yes	156 (76.8)	131 (78.0)	25 (71.4)
No	44 (21.7)	35 (20.8)	9 (25.7)
Unknown	3 (1.5)	2 (1.2)	1 (2.9)
Diabetes, *N* (%)			
Type I	3 (1.5)	3 (1.8)	0 (0)
Type II	53 (26.1)	45 (26.8)	8 (22.9)
No	147 (72.4)	120 (71.4)	27 (77.1)
Smoking status, *N* (%)			
Never	71 (35.0)	60 (35.7)	11 (31.4)
Current	26 (12.8)	19 (11.3)	7 (20.0)
Quit	104 (51.2)	88 (52.4)	16 (45.7)
Unknown	2 (1.0)	1 (0.6)	0 (0)

Note. SD: standard deviation. CHRC: Cardiac Health and Rehabilitation Centre (Hamilton Health Sciences). CHRP: Cardiac Health and Rehabilitation Program (Niagara Health Services). CABG: coronary artery bypass graft. PCI: percutaneous coronary intervention. CHF: congestive heart failure. Scores for continuous variables are represented by means and standard deviations. Scores for categorical variables are represented by percentages. No significant differences were found between sites (*P* > .05).

**Table 2:** Results from the latent class growth analyses for the moderate-strenuous physical activity.

Model	Coefficient	BIC	BLRT	Entropy
*Analysis 1 *				
Class 1 (*n* = 203)				
Intercept	7.42***			
Linear trend	12.73***			
Quadratic trend	−3.60***	5025.64	—	—

*Analysis 2 *				
Class 1 (*n* = 162)				
Intercept	3.70*			
Linear trend	9.41***			
Quadratic trend	−2.61***			
Class 2 (*n* = 41)				
Intercept	21.01**			
Linear trend	20.30*		1 versus 2 classes	
Quadratic trend	−5.648**	4909.16	137.74***	.80

*Analysis 3 *				
Class 1 (*n* = 145)				
Intercept	2.06***			
Linear trend	9.52**			
Quadratic trend	−2.60**			
Class 2 (*n* = 40)				
Intercept	29.02***			
Linear trend	1.56			
Quadratic trend	−0.97			
Class 3 (*n* = 18)				
Intercept	1.52			
Linear trend	43.72		2 versus 3 classes	
Quadratic trend	−11.40	4806.96	68.14***	.85

*P < .05; **P < .01; ***P < .001.

**Table 3:** Descriptive statistics and chi-square comparisons by class.

Patient characteristics	Inactive relapsers	Moderately active relapsers	*χ* ^ 2^
	Class 1	Class 2	
Demographics			
Age			
<65 yrs	74.8	25.2	
≥65 years	85.9	14.1	3.84
Education			
<Grade 12	82.4	17.6	
≥Grade 12	77.2	22.8	.83
Marital status			
Other	79.7	20.3	
Married/common-law	79.8	20.2	.05
Employed			
Not employed	84.1	15.9	
Employed	67.3	32.7	6.77*
Living arrangement			
Alone	83.0	17.0	
With others	78.7	21.3	.41
Clinical			
Diagnosis-MI			
No	82.9	17.1	
Yes	63.6	36.4	6.39*
NonSmoker			
No	79.4	20.6	
Yes	80.3	19.7	.02
Comorbidities			
0	61.5	38.5	
≥1	81.1	18.9	2.87

Note. *P < .01; *χ* ^2^: chi-square; MI: acute myocardial infarction. All values are in percentages.
